# Hypoxia-Preconditioned Extracellular Vesicles from Mesenchymal Stem Cells Improve Cartilage Repair in Osteoarthritis

**DOI:** 10.3390/membranes12020225

**Published:** 2022-02-16

**Authors:** Bocheng Zhang, Xiaoyuan Tian, Zhenan Qu, Jun Hao, Weiguo Zhang

**Affiliations:** 1 Second Affiliated Hospital, Dalian Medical University, Dalian 116000, China; zbocheng@outlook.com (B.Z.); tianxiaoyuany@163.com (X.T.); quzhenan2022@163.com (Z.Q.); 2 Department of Orthopaedics, First Affiliated Hospital, Dalian Medical University, Dalian 116000, China; haojundmu@163.com; 3 Graduate School, Dalian Medical University, Dalian 116000, China

**Keywords:** extracellular vesicles, mesenchymal stem cell, osteoarthritis, exosome, microRNA

## Abstract

In the past decade, mesenchymal stem cells (MSCs) have been widely used for the treatment of osteoarthritis (OA), and extracellular vesicles (EVs) may play a major role in the efficacy of this treatment. Hypoxia can change the cargo and biological functions of MSC-derived EVs (MSC-EVs). The aim of the present study was to determine whether the effects of hypoxia-preconditioned MSC-EVs on OA cartilage repair are superior to normoxia-preconditioned MSC-EVs. By using in vitro and in vivo OA models, we verified that hypoxia-preconditioned MSC-EVs improved chondrocyte proliferation and migration and suppressed chondrocyte apoptosis to a greater extent than normoxia-preconditioned MSC-EVs. Furthermore, we found that hypoxia altered the microRNA expression in MSC-EVs and identified four differentially expressed microRNAs: hsa-miR-181c-5p, hsa-miR-18a-3p, hsa-miR-376a-5p, and hsa-miR-337-5p. Bioinformatics analysis revealed that hypoxic pretreatment may promote cartilage repair by stimulating chondrocyte proliferation and migration and suppressing chondrocyte apoptosis through the miRNA-18-3P/JAK/STAT or miRNA-181c-5p/MAPK signaling pathway. Therefore, hypoxia-preconditioned EVs may be a novel treatment for OA.

## 1. Introduction

Osteoarthritis (OA) is the most common joint disease and approximately 250 million people worldwide suffer from OA; large sums of money have been invested in OA research and treatment [1]. As a total joint disease, the articular cartilage, synovium, ligament, and muscle tissue around the joint are also involved in the disease progression. The most important pathological change is articular cartilage damage. Cartilage has a limited ability to heal itself, so it cannot be repaired effectively after being damaged. At present, the first-line treatment for OA is nonsteroidal anti-inflammatory drugs (NSAIDs), but in the clinic, the relief of severe pain is not obvious and these drugs are also associated with risks of digestive tract or cardiovascular diseases [2]. Except for pain relief and anti-inflammatory effects, none of these drugs can prevent or reverse cartilage damage in OA. Therefore, it is important to develop a new treatment strategy. In the past decade, cell therapy has received much attention, especially research on mesenchymal stem cells (MSCs). As pluripotent stem cells, MSCs have the potential for directed differentiation into multiple lineages, including mesenchymal and non-mesenchymal lineages [3]. In addition to their differentiation abilities, MSCs also have a strong paracrine ability and can secrete a large number of molecules or vesicles. Recent studies have shown that the paracrine effects of MSCs are more important than differentiation in tissue repair, and more interest has been focused on extracellular vesicles (EVs) and their cargos [4].

EVs are small nanoparticles with a bimolecular membrane structure that are secreted by cells that can be found in most body fluids, such as blood, urine, and milk. Generally, according to their biological origins, EVs are mainly divided into two types: exosomes and microvesicles [5]. EVs carry a large number of factors, such as proteins, lipids, and nucleic acids, which play important roles in cell signal transmission. Among these cargos, microRNAs (miRNAs) play important roles in the biological activities of EVs, such as participating in the occurrence and development of tumors and promoting tissue repair. Furthermore, due to the bimolecular membrane structure, EV cargos are protected against enzymatic degradation in body fluids and can be stored at low temperatures. MSC-derived EVs (MSC-EVs) have been shown to have similar biological effects as MSCs, such as anti-inflammatory effects and immune regulation [6]. In recent years, emerging research on the transplantation of MSC-EVs to treat OA has obtained good results. Therefore, MSC-EVs are considered ideal substitutes for MSCs as a cell-free OA treatment [7].

In clinical applications, many MSCs need to be expanded. However, during the expansion process, MSCs are subject to technical limitations of cell death and senescence [8], but it is possible to overcome these limitations by improving the culture conditions. In the body, MSCs exist in a hypoxic environment (bone marrow is 1–9% [9], adipose tissue is 5–9% [10], and umbilical cord blood is 1–6% [11]), whereas in vitro, MSCs are exposed to 21% oxygen in culture. In vitro experiments have demonstrated that a hypoxic environment improves the proliferation and migration of MSCs during the expansion process as well as promotes the differentiation ability [12,13]. Similarly, hypoxia can also change the expression of EV contents and affect biological functions [14,15]. Hypoxia improves the angiogenic effects of MSC-EVs by changing the expression of miRNAs and proteins [16]. However, the role of hypoxia-preconditioned MSC-EVs in OA is still unclear. In this study, we hypothesized that EVs that are secreted by hypoxia-pretreated MSCs could exert increased therapeutic effects on OA. In vitro studies confirmed that MSC-EVs could improve the proliferation, migration, and apoptosis of inflammatory chondrocytes. Next, in vitro and in rat OA models, hypoxic pretreatment was confirmed to further enhance the therapeutic effect. Finally, the mechanism was explored through miRNA sequencing and pathway enrichment analyses, and the effective components and signaling pathways through which hypoxia alters the efficacy of OA treatment were further determined.

## 2. Materials and Methods

### 2.1. Animals

Sprague-Dawley rats that were four-weeks-old were used for this study. All the animal experimental protocols were approved by the Animal Investigation Committee of Dalian Medical University.

### 2.2. Isolation and Culture of Chondrocytes

To obtain chondrocytes, the knee, hip, and shoulder joints of rats were isolated. The soft tissue around the joint was removed with ophthalmic scissors and the cartilage tissue was cut into small pieces (approximately 1 mm in diameter) and digested with 0.2% type II collagenase (Sigma-Aldrich, Burlington, MA, USA) for 8 h at 37 °C. The medium was filtered and collected into a centrifuge tube and then centrifuged at 1500× *g* for 5 min. The cell pellet was resuspended after centrifugation, and the cells were plated in culture medium (DMEM/F-12 medium that was supplemented with 10% fetal bovine serum (FBS, Gibco, Burlington, MA, USA) and 1% penicillin-streptomycin (P/S, Sigma-Aldrich, USA)) at a density of 2 × 10^4^/cm^2^ and incubated in 5% CO_2_ at 37 °C. The medium was replaced every 3 days and passage 1 (P1) cells were used for subsequent experiments.

### 2.3. Culture and Characterization of Bone Marrow MSCs (BMSCs)

Adult BMSCs were purchased from Cyagen Biosciences (Guangzhou, China) and cultured in complete growth medium (DMEM/F-12 medium that was supplemented with 10% FBS and 1% P/S) at 37 °C in a 5% CO_2_ cell culture incubator, and the adherent cells were passaged after reaching 70–80% confluence. P4 cells were used for subsequent experiments.

For phenotypic characterization, BMSCs were stained with mouse anti-human polyclonal antibodies against CD14, CD19, CD45, CD90, CD105, and CD73 (BD Pharmingen, San Diego, CA, USA) and analyzed by flow cytometry (BD Biosciences, Franklin Lakes, NJ, USA). The data were analyzed using FlowJo^TM^ V10 software.

For multidirectional differentiation potential analysis, osteogenic induction medium, adipogenic induction medium, and chondrogenic induction medium (all from Cyagen, Suzhou, China) were used to replace the complete medium and induce differentiation. The process of inducing differentiation was performed as described in the manufacturer’s instructions and then assessed by Alizarin red staining, Oil Red O staining, and Alimin blue staining.

### 2.4. Preparation and Identification of EVs

BMSCs were cultured in normal oxygen cell incubators at 37 °C with 5% CO_2_ and 21% O_2_ or in hypoxic cell incubators with 5% O_2_ in DMEM/F12 containing 10% EV-depleted FBS (Vivacell, Shanghai, China) for 48 h. The medium was collected and centrifuged at 3000× *g* for 10 min to remove the dead cells and then centrifuged at 10,000× *g* for 30 min to remove the cell debris. The supernatant was ultracentrifuged at 120,000× *g* at 4 °C for 1 h twice. The pellets were resuspended in phosphate-buffered saline (PBS) and then the protein concentration was determined [17]. EVs were used immediately or stored at −80 °C.

NanoSight tracking analysis (NTA, Malvern, UK) was used to measure the concentration and size distribution of EVs. Transmission electron microscopy (TEM; Joel, Tokyo, Japan) was used to identify EV morphology. Surface marker expression of the signature EV proteins TSG101 (1:1000, Proteintech), CD9 (1:1000, Abcam), and HSP70 (1:1000, Proteintech) was determined by Western blotting.

EVs were fluorescently labeled with PKH67 (PKH67GL-1KT, Sigma, GER Burlington, MA, USA) according to the manufacturer’s instructions. The labeled EVs were incubated with chondrocytes for 12 h. The chondrocytes were fixed with 4% paraformaldehyde, mounted using mounting medium containing DAPI (Abcam, USA), and observed under a laser confocal microscope (Leica, GER Burlington, MA, USA).

### 2.5. Cell Proliferation Assay

The Cell Counting Kit 8 (CCK-8) assay (Dojindo, Tokyo, Japan) was used to measure cell proliferation. Briefly, the chondrocytes were seeded in a 96-well plate at a density of 2000 cells/well. Next, 10 ng/mL IL-1β (PeproTech, Rocky Hill, NJ, USA) was added to induce inflammation, while the control cells were not stimulated. After 24 h of modeling, the medium was replaced with fresh medium containing different concentrations of EVs (5, 10, 20 μg/mL). Next, 10 μL of CCK-8 solution was added and the samples were incubated for 2 h in the incubator. A microplate reader (Infinite f50, TECAN, Basel, Switzerland) was used to measure the optical density (OD) at a wavelength of 450 nm.

### 2.6. Cell Migration Assay

A Transwell assay was used to analyze the effect of EVs that were secreted by MSCs on the migration of chondrocytes. In brief, after being digested and counted, approximately 5 × 10^4^ chondrocytes were seeded into the upper chambers of a 24-well Transwell plate with 8-μm pores (Corning, Corning, NY, USA). Next, 600 μL of complete chondrocyte culture medium containing various concentrations of EVs was added to the lower chambers of the Transwell plate and incubated for 12 h at 37 °C. The upper chamber was then fixed with 4% paraformaldehyde for 15 min, stained with 0.5% crystal violet for 10 min and washed with PBS three times. The upper surface of the upper chamber was carefully wiped using a cotton swab to remove the cells that had not migrated to the lower surface. A total of five randomly selected fields per well were photographed using a microscope (Olympus, Tokyo, Japan) and assessed by two pathologists in a blinded manner.

### 2.7. Cell Apoptosis Assay

We used an Annexin V apoptosis detection kit (BD Pharmingen™, San Diego, CA, USA) to measure apoptosis in inflammatory chondrocytes. After treatment with different concentrations of EVs for 48 h, the chondrocytes were digested, washed, and resuspended in binding buffer. Annexin V and propidium iodide (PI) were added and the samples were incubated in the dark for 15 min at room temperature. The cells that were labeled with fluorescent dyes were analyzed by flow cytometry within 30 min.

### 2.8. miRNA Sequencing and Bioinformatics Analysis

The extraction of tRNA from EVs, construction of an miRNA library, and miRNA sequencing were performed by LC-BIO Technologies (Hangzhou, China). Standard procedures that were provided by Illumina were followed, including those for library preparation and sequencing. Small RNA sequencing libraries were prepared using TruSeq Small RNA Sample Prep Kits (Illumina, San Diego, CA, USA). After library preparation, the constructed library was sequenced using an Illumina HiSeq 2000/2500. We obtained valid data and compared precursors and genomes to identify miRNAs and performed differential analysis of miRNAs. The expression levels of miRNAs with a *p*-value < 0.05 were considered to indicate a significant difference. Among miRNAs with significant differences, a fold change (FC) greater than 2 or less than 0.5 was defined as differentially expressed miRNAs. We used the GSE156919 dataset in the GEO database to verify the differentially expressed miRNAs.

There were two miRNA target-predicting algorithms (mirdb and TargetScan) that were used to identify the potential downstream targets of the differentially expressed miRNAs. To examine the underlying functions of the selected miRNAs and target mRNAs, Kyoto Encyclopedia of Genes and Genomes (KEGG) pathway enrichment analysis and Gene Ontology (GO) term analysis were performed using the KOBAS 3.0 (http://kobas.cbi.pku.edu.cn/, accessed on 15 July 2021) and DAVID 6.8 (https://david.ncifcrf.gov/, accessed on 15 July 2021) databases. KEGG pathway analysis was performed to identify the pathways that related to miRNAs and target mRNAs. GO analysis assessed the molecular functions (MFs), biological processes (BPs), and cellular components (CCs). The target genes were introduced into the STRING 11.0 (https://www.string-db.org/, accessed on 15 July 2021) database to evaluate the functional correlations between them, and, to identify hub genes, the connectivity extent in the protein-protein interaction (PPI) network was investigated through Cytoscape 3.7.1.

### 2.9. OA Model Induction and EV Administration

Female Sprague-Dawley rats (4-weeks-old) were housed in a specific pathogen-free (SPF) animal laboratory with a 12:12-h light/dark cycle, a controlled temperature environment (23–25 °C), and a steady humidity were used in these studies. The modified Hulth method was used to induce the OA model [18]. The rats were anesthetized by an intraperitoneal injection of pentobarbital (50 mg/kg). Following anesthetization, a 2-cm longitudinal incision was made on the medial side of the right knee joint; the anterior cruciate ligament was cut, the medial meniscus was eliminated, and suturing was performed layer by layer. After surgery, all the rats received 5 mg/kg gentamicin. All the rats were randomly divided into four groups (*n* = 10/group): (1) Control group; (2) OA group; (3) OA + Normoxia-EV group; and (4) OA + Hypoxia-EV group. During the 4th week after surgery, the rats were injected with EVs (100 μg of total EV protein in 100 μL of PBS) or an equivalent volume of PBS (100 μL). A total of eight weeks after surgery, the rats were sacrificed by anesthetic overdose and knee samples were harvested to evaluate disease progression.

### 2.10. Histological Staining

After being fixed with paraformaldehyde for 24 h and decalcified for 1 month in EDTA decalcifying solution (Solarbio, Beijing, China), the samples were embedded in paraffin and sectioned into 5-μm-thick sections. Serial sections were obtained from the medial and lateral compartments at 200-μm intervals. The sections were dewaxed and rehydrated before the experiments. Saffron O-fast green (Solarbio, Beijing, China) was used for histological staining, and the Osteoarthritis Research Society International (OARSI) scores were calculated to evaluate the degeneration of the articular cartilage. The stained sections were randomly assigned to two different pathologists for evaluation in a blinded manner.

### 2.11. Statistical Analysis

Each experiment was performed with at least 3 biological replicates. All data are presented as the mean ± standard deviation (SD) and were analyzed and displayed with SPSS 25.0 software (IBM Corporation, New York, NY, USA). The independent *t*-test was used to identify differences between groups as appropriate. A one-way analysis of variance (ANOVA) was carried out for multiple group comparisons. Statistical significance was determined at the level of *p* < 0.05.

## 3. Results

### 3.1. Characterization of BMSCs and BMSC-EVs

**Figure 1:**
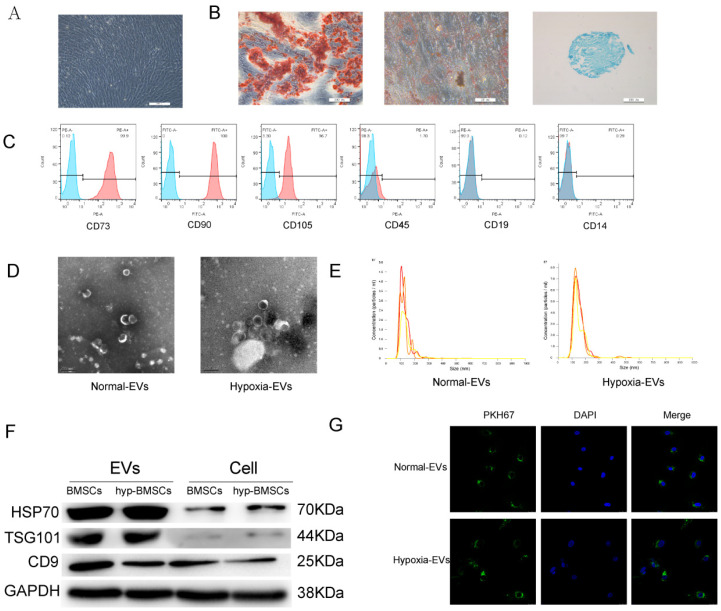
Identification of BMSCs and BMSC-EVs. (**A**) BMSCs showed a spindle-like shape (Scale bar = 200 µm). (**B**) BMSCs were induced to differentiate into osteoblast (Scale bar = 200 µm), adipose cells (Scale bar = 50 µm), and chondroblast cells (Scale bar = 200 µm). (**C**) Flow cytometry analysis of the cell surface markers on BMSCs. (**D**) Morphology of the exosomes under transmission electron microscopy. (Scale bar = 200 nm). (**E**) Particle size distribution of BMSC-EVs as measured by Nanosight. (**F**) Western blot analysis of the EV-specific CD9, CD70, and TSG101 proteins. (**G**) Detection of BMSC-EVs uptake by chondrocytes. PKH67 is shown in red, and DAPI is shown in blue (Scale bar = 20 µm).

P4 BMSCs were observed under an optical microscope, and the image in Figure 1A shows that the cells displayed typical MSC morphology with a spindle-like shape. To identify the multilineage differentiation potential of these cells, BMSCs were cultured in osteogenic-, chondrogenic-, and adipogenic-induction medium for 4 weeks. Alizarin red, oil red O, and Alimin blue staining results confirmed that BMSCs had multidirectional differentiation potential and formed osteoblasts, adipocytes, and chondrocytes (Figure 1B). Flow cytometry analysis showed that the BMSCs were positive for mesenchymal markers, including CD73, CD90, and CD105, but negative for CD45, CD19, and CD14 (Figure 1C).

EVs were isolated from the BMSC supernatant by ultracentrifugation. The purified BMSC-EVs were identified by TEM, NTA, and Western blotting. TEM showed that the EVs exhibited an oval shape (Figure 1D). The NTA revealed that the average vesicle diameter was approximately 150 nm (Figure 1E). Western blot analysis of EV lysates revealed a positive expression of the EV surface markers HSP70, TSG101, and CD9 (Figure 1F). Collectively, these results confirm the successful isolation of EVs from the supernatant of BMSCs. We performed these analyses on hypoxia-derived EVs, and the results showed that BMSCs can also secrete EVs in a hypoxic environment.

To investigate the feasibility of using BMSC-EVs to treat OA, we examined the cellular uptake of PKH67-labeled EVs. After the chondrocytes were incubated with PKH67-labeled EVs for 12 h, EVs were observed in the cytoplasm by fluorescence microscopy to confirm uptake (Figure 1G).

### 3.2. BMSC-EVs Relieve OA

**Figure 2:**
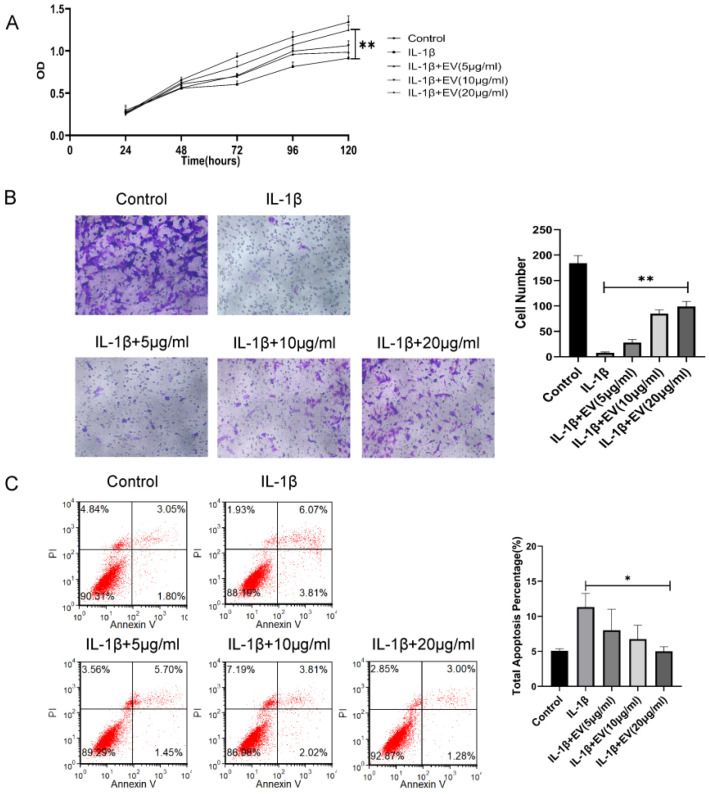
BMSC-EVs attenuated the proliferation and migration and inhibited the apoptosis of inflammatory chondrocytes. (**A**) The proliferation of chondrocytes was evaluated by CCK-8 assay. (**B**) Chondrocyte migration was determined by the Transwell migration assay (Scale bar = 50 µm). (**C**) Flow cytometry was used to analyze apoptosis in inflammatory chondrocytes. * *p* < 0.05, ** *p* < 0.01.

Next, the effect of EVs on the chondrocytes was evaluated. IL-1β is an important factor in the development of cartilage degeneration in OA, and IL-1β was added to simulate the OA inflammatory environment. We treated inflammatory chondrocytes with different concentrations of BMSC-EVs (5, 10, and 20 µg/mL). The CCK-8 assay results indicated that IL-1β treatment significantly reduced the proliferation of chondrocytes and EVs, especially at 20 μg/mL, and significantly attenuated the IL-1β-induced inhibition of chondrocyte proliferation (Figure 2A). The effects of EVs on the apoptosis rate of inflammatory chondrocytes were analyzed by Annexin V/PI staining. The results suggested that EVs could reduce the apoptosis rate of inflammatory chondrocytes (Figure 2B). Similarly, inflammatory chondrocyte migration was significantly attenuated in response to increasing EV concentrations (Figure 2C). In summary, these findings demonstrated that BMSC-EVs improve the apoptosis, migration, and proliferation of inflammatory chondrocytes and that the effects are optimized as the concentration is increased.

### 3.3. Hypoxia-EVs Have Improved Therapeutic Effects

**Figure 3:**
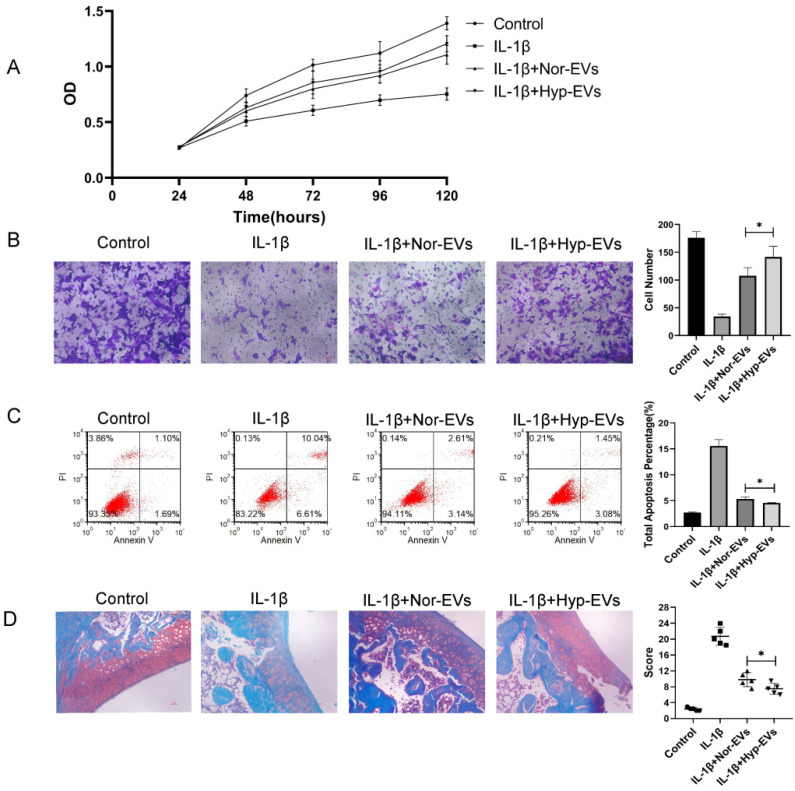
Hypoxia preconditioning improved the therapeutic effects of BMSC-EVs in vitro and in vivo. (**A**) The proliferation of chondrocytes was evaluated by CCK-8 assay. (**B**) Chondrocyte migration was determined by the Transwell migration assay (Scale bar = 50 µm). (**C**) Flow cytometry was used to analyze apoptosis in inflammatory chondrocytes. (**D**) Safranin O and fast green staining of knee joint specimens of rats that were treated with normoxia-EVs or hypoxia-EVs. Statistical analysis of the OARSI score in rats treated with normoxia-EVs or hypoxia-EVs. * *p* < 0.05.

To explore the effect of hypoxia on the efficacy of BMSC-EVs in the treatment of inflammatory chondrocytes, we assessed the effect of hypoxia-EVs via CCK-8 assays, Transwell assays, and apoptosis assays. The CCK-8 assay results revealed that, compared to that of the IL-1β group, chondrocyte proliferation increased in the normoxia-EV and hypoxia-EV groups. However, hypoxia-EV administration caused a further increase in proliferation (Figure 3A). Next, we used a Transwell assay to determine the effect of hypoxia-EVs on chondrocyte migration. Compared to normoxia-EVs, hypoxia-EVs greatly enhanced the migration ability of chondrocytes (Figure 3B). Similarly, the apoptosis assay showed that both the hypoxia group and the normoxia group had reduced apoptosis rates, and the effect of treatment on the hypoxia group was more obvious than that in the other groups (Figure 3C). Overall, these findings indicate that, compared to normoxic treatment, hypoxic pretreatment can improve the therapeutic efficacy of BMSC-EVs on inflammatory chondrocytes to a greater extent.

Next, to verify the chondroprotective effect of hypoxia-EVs in vivo, a rat OA model was used. At 4 weeks after surgery, 100 μL of PBS, normoxia-EV (50 μg/100 μL), and hypoxia-EVs (50 μg/100 μL) were injected into the joint cavity. After four weeks following joint injection, we collected joint tissues for pathological evaluation. In sections that were stained with safranin O-fast green (Figure 3D), a reduction in safranin O staining was noted in the normoxia-EV group compared with the hypoxia-EV group, which indicated a loss of proteoglycan in the cartilage in the normoxia-EV group. The OARSI scores in the control, normoxia-EV, and hypoxia-EV groups were significantly lower than those in the OA group (Figure 3D). The OARSI score of the hypoxia-EV group was significantly lower than that of the normoxia-EV group.

### 3.4. Profiling Differentially Expressed miRNAs

**Figure 4:**
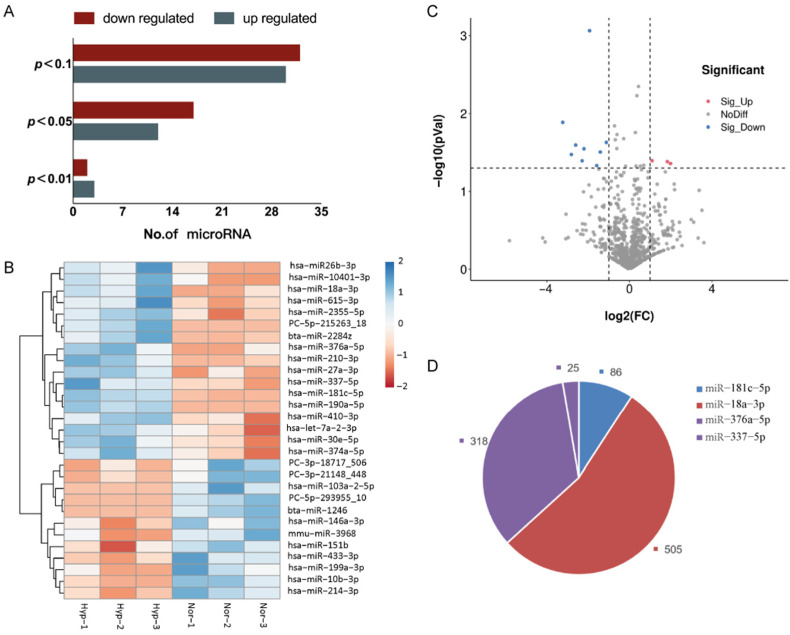
Hypoxia regulates miRNAs in BMSC-EVs. (**A**) The number of differentially expressed miRNAs. There were 29 differentially expressed miRNAs with a *p*-value < 0.05. (**B**) A heatmap of the 29 differentially expressed miRNAs. (**C**) Volcano plot displaying the 12 significant differentially expressed miRNAs based on the criteria of *p* < 0.05 and FC > 2 or <0.5. (**D**) Target gene prediction of 4 potential differentially expressed miRNAs. (913 possible target genes of these 4 miRNAs were predicted by using mirdb and TargetScan).

Using a significance threshold of *p* < 0.05, a total of 29 differentially expressed miRNAs were identified in the exposure group, of which 12 miRNAs were upregulated and 17 miRNAs were downregulated (Figure 4A,B). The upregulated miRNAs were hsa-miR-433-3p, hsa-miR-199a-3p, hsa-miR-214-3p, hsa-miR-10b-3p, hsa-miR-151b, mmu-miR-3968, PC-3p-18717, hsa-miR-146a-3p, PC-3p-21148, PC-5p-293955, bta-miR-1246, and hsa-miR-103a-2-5p, while the downregulated miRNAs were hsa-miR-181c-5p, hsa-miR-190a-5p, hsa-miR-18a-3p, hsa-miR-2355-5p, PC-5p-215263, hsa-miR-27a-3p, hsa-miR-410-3p, hsa-miR-30e-5p, hsa-miR-337-5p, hsa-miR-376a-5p, hsa-miR-374a-5p, hsa-miR-210-3p, bta-miR-2284z, hsa-miR-26b-3p, hsa-miR-10401-3p, hsa-let-7a-2-3p, and hsa-miR-615-3p.

Based on the criteria of *p* < 0.05 and FC > 2 or <0.5, a total of 12 significant differentially expressed miRNAs were identified by miRNA sequencing and selected for validation by the GSE156919 dataset in the GEO database (Figure 4C). Consistent with the miRNA sequencing results and the GSE156919 dataset, the validation data showed that hsa-miR-181c-5p (FC = 0.26, *p* < 0.001), hsa-miR-18a-3p (FC = 0.11, *p* < 0.05), hsa-miR-376a-5p (FC = 0.16, *p* < 0.05), and hsa-miR-337-5p (FC = 0.46, *p* < 0.05) were downregulated in hypoxia-EVs; a total of 913 possible target genes of these 4 miRNAs (931 possible target genes, including 18 duplicates) were predicted by using mirdb and TargetScan (Figure 4D).

### 3.5. Target Annotation

**Figure 5:**
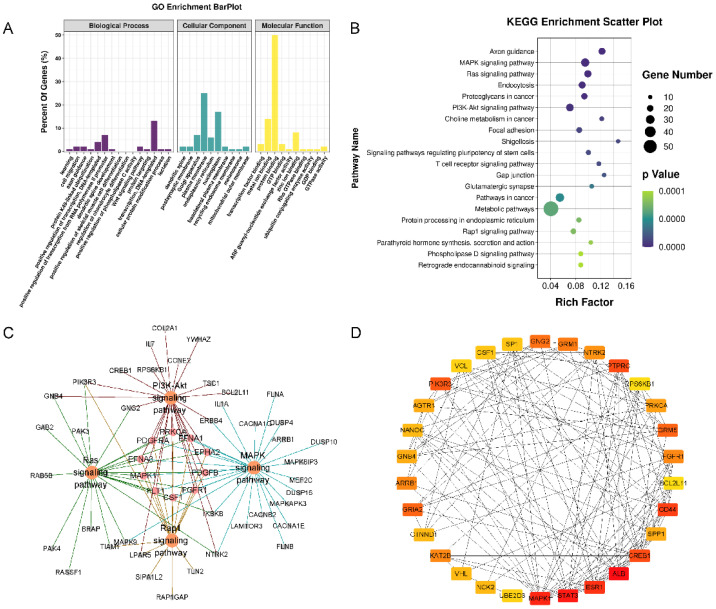
Functional enrichment analysis of the target genes of 4 miRNAs. (**A**) GO pathway enrichment analysis of the target genes of 4 miRNAs. (**B**) KEGG pathway enrichment analysis of the target genes of 4 miRNAs. (**C**) The target-gene regulatory network of MAPK, RAS, PI3K-Akt, and Rap1 signaling pathways. (**D**) The PPI networks of the top 30 hub target genes. ALB, STAT3, and MAPK1 exhibited the highest node degrees (62, 58, and 54, respectively).

Three types of annotations for GO functions were conducted for possible target genes. The various GO functions are shown in Figure 5A and include cell migration (GO:0016477), regulation of chondrocyte differentiation (GO:0032330), Wnt signaling pathway (GO:0016055), and apoptotic process (GO:0006915) in the BP category; Golgi apparatus, plasma membrane endoplasmic reticulum, and nucleoplasm were identified in the CC category; and transcription factor binding, protein binding, and DNA binding were identified in the MF category. KEGG pathway enrichment analysis was performed to investigate the pathways of these target genes, including the MAPK, RAS, PI3K-Akt, and Rap1 signaling pathways (Figure 5B,C).

The PPI networks of the target genes were determined using the STRING database. The data indicated that a number of target genes were able to interact with each other. The top 30 hub genes were selected based on the node degree (Figure 5D). ALB, STAT3, and MAPK1 exhibited the highest node degrees (62, 58, and 54, respectively), indicating that these genes were potentially important target genes that were related to OA.

## 4. Discussion

OA is a total joint disease in which the main pathological change is damage to articular cartilage. OA is characterized by anabolic and catabolic disorders of the ECM of articular cartilage. Furthermore, studies have shown that promoting chondrocyte proliferation and migration and inhibiting cell apoptosis are key points in OA treatment. Our results indicated that BMSC-EVs can promote cartilage repair through different mechanisms and that hypoxic pretreatment can enhance the therapeutic effects. The sequencing results showed that hypoxia can change the expression of miRNAs, and subsequent bioanalysis results showed that these differentially expressed miRNAs play important roles in OA.

At present, many clinical studies using MSCs to treat OA have achieved certain therapeutic effects [19,20]. However, there are still some problems; for example, due to technical limitations, there are certain difficulties in obtaining and preserving cells. As a donor ages, the number and function of MSCs also decline [21]. It was also found that MSCs are short-lived after systemic administration. Although MSCs have a certain ability to differentiate, studies have shown that the paracrine function of MSCs, especially the secretion of EVs, plays a major role in tissue repair. At present, many preclinical studies have achieved therapeutic effects by using MSC-EVs to treat different diseases, including OA [22]. It is well known that there is a large difference between the oxygen concentration in vivo and in vitro. In vitro cell culture generally requires 21% oxygen, and in vivo MSCs exist at oxygen levels of approximately 5% [23]. Furthermore, studies have confirmed that hypoxia can change the physiological functions of MSCs, such as promoting chondrogenic differentiation, and change the function of MSC-EVs. EVs that are secreted by MSCs at less than 5% oxygen promote angiogenesis in animal models of myocardial infarction and fracture better than EVs that are secreted by MSCs under normoxic conditions [24,25]. However, the role of hypoxia-preconditioned MSC-EVs in OA is still unclear. Therefore, we hypothesized that hypoxia could promote the cartilage regeneration abilities of MSC-EVs, verified this hypothesis through in vivo and in vitro experiments, and finally explored the underlying mechanism through sequencing.

The TEM and NTA results did not show any morphological differences between the normoxic and hypoxic MSC-EV groups in terms of size, shape, or electron density. Previous studies have shown that hypoxic pretreatment increases the ability of MSCs to secrete EVs. In the present study, in vitro experiments showed that EVs promoted chondrocyte proliferation and migration and suppressed chondrocyte apoptosis and that these therapeutic effects were enhanced by hypoxic pretreatment. In the animal model of OA, the hypoxia group also showed a better therapeutic effect than the other groups.

As we observed the beneficial effects of normoxia-EVs compared to hypoxia-EVs, we focused on identifying the underlying mechanisms that caused this difference between normoxia- and hypoxia-EVs. miRNAs are short RNA molecules that regulate multiple cellular processes through posttranscriptional gene silencing. Similarly, miRNAs in EVs play an important role in different BPs, and external stimuli can change the expression of miRNAs. Many studies have confirmed that hypoxia changes the expression of miRNAs, such as miR-210 [26,27], miR-126 [28], and miR-22 [29], in MSC-EVs, further changing their biological functions. Through miRNA sequencing, we found that hypoxia pretreatment could change the expression of miRNAs, and a total of 12 miRNAs were significantly changed. Subsequently, we used the GSE156919 dataset to verify these differentially expressed miRNAs and found four miRNAs with the same expression trend: hsa-miR-181c-5p, hsa-miR-18a-3p, hsa-miR-376a-5p, and hsa-miR-337-5p. Several recent studies have reported that these four miRNAs are related to OA [30]. The decrease in miR-181 reduces the expression of TNF-α and IL-6 by downregulating the NF-κB signaling pathway, thus repressing the occurrence of OA [31].

In total, 913 possible target genes were predicted for the four miRNAs by using miRDB and the TargetScan database. GO and KEGG enrichment analyses were performed to explore the interactions among the target genes, which were mainly enriched in BP terms that were associated with cell migration, the regulation of chondrocyte differentiation, the Wnt signaling pathway and apoptotic processes, in the MAPK signaling pathway, RAS signaling pathway, PI3K-Akt signaling pathway, and Rap1 signaling pathway. These BPs and pathways are likely to be involved in the progression of OA and cartilage repair. In the PPI network, ALB, STAT3, and MAPK1 had the highest node degrees and were selected as hub genes. Numerous studies have demonstrated that these three genes or proteins are important molecules that are associated with OA.

ALB, also known as albumin, encodes the most abundant protein in human blood. ALB is the main component of synovial fluid. As OA progresses, the level of ALB also decreases. ALB has been reported to induce anti-inflammatory effects and upregulate the transcription of collagen Ⅱ during chondrogenic differentiation, thereby affecting OA treatment [32]. Currently, some clinical trials of the use of ALB to treat OA have achieved good results, and there were no severe drug-related side effects during treatment. STAT3, a member of the STAT protein family, mediates the expression of a variety of genes in response to cell stimuli and plays a key role in many cellular processes, such as cell growth and apoptosis. STAT3 forms part of the JAK-STAT signal cascade, which is the basis of many cytokine receptor signal transduction mechanisms [33]. The JAK-STAT3 signaling pathway is involved in metabolism, proliferation, apoptosis, and inflammation in OA [34]. MAPK1, also known as ERK, belongs to the MAPK family and is related to various cell processes, including proliferation, differentiation, transcription modulation, and development. MAPK is a mediator that regulates the downstream expression of proinflammatory cytokines and MMPs [35]. Numerous studies have demonstrated that MAPK1 is an important molecule that is associated with OA, and miRNA-186-5p downregulation inhibits OA development by targeting MAPK1 [36].

**Figure 6:**
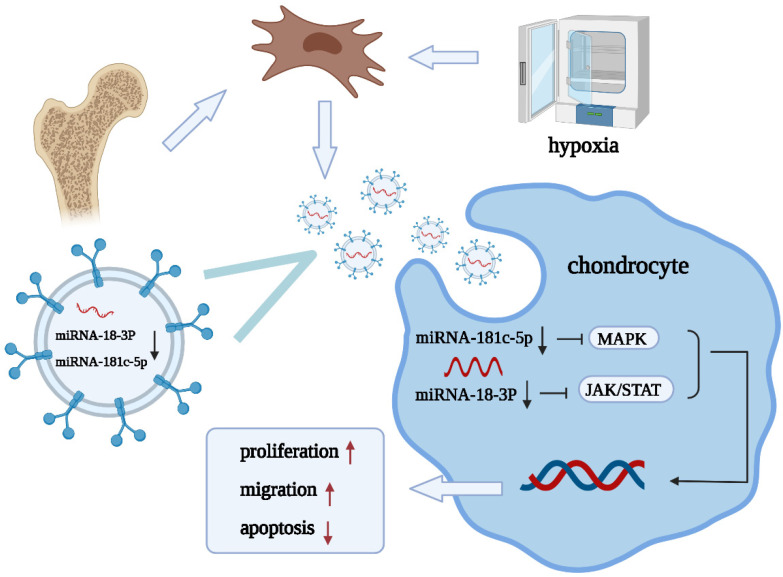
Diagram illustrating the proposed mechanism of action of hypoxia-preconditioned BMSC-EVs in OA.

Our results showed that hypoxic pretreatment could reduce the expression of miR-181c-5p, miR-18a-3p, miR-376a-5p, and miR-337-5p in EVs, and their target genes, such as ALB, STAT3, and MAPK1, are related to OA and cartilage repair. Based on these experimental results, we propose the following mechanism: hypoxia-derived EVs that are derived from BMSCs promote cartilage repair through the miRNA-18-3P/JAK-STAT or miRNA-181c-5p/MAPK signaling pathways (Figure 6). However, the present study has several limitations; (1) we studied only the changes in miRNAs and did not focus on the changes in other cargos within EVs, and (2) studies on the specific mechanisms underlying the modulation of OA by miR-181c-5p, miR-18a-3p, miR-376a-5p, and miR-337-5p were limited.

## 5. Conclusions

We verified that BMSC-EVs promote proliferation and migration and inhibit apoptosis during cartilage repair and that hypoxic pretreatment improves these repair functions. This improvement may be due to changes in miRNA expression, and the underlying mechanism may involve the miRNA-18-3P/JAK-STAT or miRNA-181c-5p/MAPK signaling pathway. In the future, miRNAs that are combined with EVs will serve as a new strategy for the treatment of OA.

## Data Availability

The data that support the findings of this study are available from the corresponding author (W.Z.) upon reasonable request.

## References

[1] Hunter D.J., Bierma-Zeinstra S. (2019). Osteoarthritis. Lancet.

[2] Katz J.N., Arant K.R., Loeser R.F. (2021). Diagnosis and Treatment of Hip and Knee Osteoarthritis: A Review. JAMA.

[3] Krampera M., Le Blanc K. (2021). Mesenchymal stromal cells: Putative microenvironmental modulators become cell therapy. Cell Stem Cell.

[4] Kalluri R., LeBleu V.S. (2020). The biology, function, and biomedical applications of exosomes. Science.

[5] Zhang B., Tian X., Hao J., Xu G., Zhang W. (2020). Mesenchymal Stem Cell-Derived Extracellular Vesicles in Tissue Regeneration. Cell Transplant..

[6] Herrmann I.K., Wood M.J.A., Fuhrmann G. (2021). Extracellular vesicles as a next-generation drug delivery platform. Nat. Nanotechnol..

[7] Zhang B., Tian X., Qu Z., Liu J., Yang L., Zhang W. (2021). Efficacy of extracellular vesicles from mesenchymal stem cells on osteoarthritis in animal models: A systematic review and meta-analysis. Nanomedicine.

[8] Derubeis A.R., Cancedda R. (2004). Bone marrow stromal cells (BMSCs) in bone engineering: Limitations and recent advances. Ann Biomed. Eng..

[9] Spencer J.A., Ferraro F., Roussakis E., Klein A., Wu J., Runnels J.M., Zaher W., Mortensen L.J., Alt C., Turcotte R. (2014). Direct measurement of local oxygen concentration in the bone marrow of live animals. Nature.

[10] Hodson L. (2014). Adipose tissue oxygenation: Effects on metabolic function. Adipocyte.

[11] Sjostedt S., Rooth G., Caligara F. (1960). The Oxygen Tension of the Blood in the Umbilical Cord and the Intervillous Space. Arch. Dis. Child..

[12] Taheem D.K., Foyt D.A., Loaiza S., Ferreira S.A., Ilic D., Auner H.W., Grigoriadis A.E., Jell G., Gentleman E. (2018). Differential Regulation of Human Bone Marrow Mesenchymal Stromal Cell Chondrogenesis by Hypoxia Inducible Factor-1alpha Hydroxylase Inhibitors. Stem Cells.

[13] Zhu C., Yu J., Pan Q., Yang J., Hao G., Wang Y., Li L., Cao H. (2016). Hypoxia-inducible factor-2 alpha promotes the proliferation of human placenta-derived mesenchymal stem cells through the MAPK/ERK signaling pathway. Sci. Rep..

[14] Yang Y., Lee E.H., Yang Z. (2021). Hypoxia conditioned mesenchymal stem cells in tissue regeneration application. Tissue Eng. Part B Rev..

[15] Liu J., He J., Ge L., Xiao H., Huang Y., Zeng L., Jiang Z., Lu M., Hu Z. (2021). Hypoxic preconditioning rejuvenates mesenchymal stem cells and enhances neuroprotection following intracerebral hemorrhage via the miR-326-mediated autophagy. Stem Cell Res. Ther..

[16] Gregorius J., Wang C., Stambouli O., Hussner T., Qi Y., Tertel T., Borger V., Mohamud Yusuf A., Hagemann N., Yin D. (2021). Small extracellular vesicles obtained from hypoxic mesenchymal stromal cells have unique characteristics that promote cerebral angiogenesis, brain remodeling and neurological recovery after focal cerebral ischemia in mice. Basic Res. Cardiol..

[17] Thery C., Amigorena S., Raposo G., Clayton A. (2006). Isolation and characterization of exosomes from cell culture supernatants and biological fluids. Curr. Protoc. Cell Biol..

[18] Rogart J.N., Barrach H.J., Chichester C.O. (1999). Articular collagen degradation in the Hulth-Telhag model of osteoarthritis. Osteoarthr. Cartil..

[19] Yan X., Yang B., Chen Y., Song Y., Ye J., Pan Y., Zhou B., Wang Y., Mao F., Dong Y. (2021). Anti-Friction MSCs Delivery system improves the therapy for severe osteoarthritis. Adv. Mater..

[20] Lamo-Espinosa J.M., Blanco J.F., Sanchez M., Moreno V., Granero-Molto F., Sanchez-Guijo F., Crespo-Cullel I., Mora G., San Vicente D.D., Pompei-Fernandez O. (2020). Phase II multicenter randomized controlled clinical trial on the efficacy of intra-articular injection of autologous bone marrow mesenchymal stem cells with platelet rich plasma for the treatment of knee osteoarthritis. J. Transl. Med..

[21] Wang S., Wang Z., Su H., Chen F., Ma M., Yu W., Ye G., Cen S., Mi R., Wu X. (2021). Effects of long-term culture on the biological characteristics and RNA profiles of human bone-marrow-derived mesenchymal stem cells. Mol. Ther. Nucleic Acids.

[22] Li S., Liu J., Liu S., Jiao W., Wang X. (2021). Mesenchymal stem cell-derived extracellular vesicles prevent the development of osteoarthritis via the circHIPK3/miR-124-3p/MYH9 axis. J. Nanobiotechnol..

[23] Buravkova L.B., Andreeva E.R., Gogvadze V., Zhivotovsky B. (2014). Mesenchymal stem cells and hypoxia: Where are we?. Mitochondrion.

[24] Qiu X., Liu J., Zheng C., Su Y., Bao L., Zhu B., Liu S., Wang L., Wang X., Wang Y. (2020). Exosomes released from educated mesenchymal stem cells accelerate cutaneous wound healing via promoting angiogenesis. Cell Prolif..

[25] Goodman S.B., Lin T. (2020). Modifying MSC Phenotype to Facilitate Bone Healing: Biological Approaches. Front. Bioeng. Biotechnol..

[26] Cheng H., Chang S., Xu R., Chen L., Song X., Wu J., Qian J., Zou Y., Ma J. (2020). Hypoxia-challenged MSC-derived exosomes deliver miR-210 to attenuate post-infarction cardiac apoptosis. Stem Cell Res. Ther..

[27] Zhu J., Lu K., Zhang N., Zhao Y., Ma Q., Shen J., Lin Y., Xiang P., Tang Y., Hu X. (2018). Myocardial reparative functions of exosomes from mesenchymal stem cells are enhanced by hypoxia treatment of the cells via transferring microRNA-210 in an nSMase2-dependent way. Artif. Cells Nanomed. Biotechnol..

[28] Liu W., Li L., Rong Y., Qian D., Chen J., Zhou Z., Luo Y., Jiang D., Cheng L., Zhao S. (2020). Hypoxic mesenchymal stem cell-derived exosomes promote bone fracture healing by the transfer of miR-126. Acta Biomater..

[29] Feng Y., Huang W., Wani M., Yu X., Ashraf M. (2014). Ischemic preconditioning potentiates the protective effect of stem cells through secretion of exosomes by targeting Mecp2 via miR-22. PLoS ONE.

[30] Huang B., Yu H., Li Y., Zhang W., Liu X. (2019). Upregulation of long noncoding TNFSF10 contributes to osteoarthritis progression through the miR-376-3p/FGFR1 axis. J. Cell Biochem..

[31] Zhu L.M., Yang M. (2019). The suppression of miR-181 inhibits inflammatory responses of osteoarthritis through NF-kappaB signaling pathway. Eur. Rev. Med. Pharmacol. Sci..

[32] Marian M., Shah R., Gashi B., Zhang S., Bhavnani K., Wartzack S., Rosenkranz A. (2021). Exploring the lubrication mechanisms of synovial fluids for joint longevity—A perspective. Colloids Surf. B Biointerfaces.

[33] Rong Y., Zhang J., Jiang D., Ji C., Liu W., Wang J., Ge X., Tang P., Yu S., Cui W. (2021). Hypoxic pretreatment of small extracellular vesicles mediates cartilage repair in osteoarthritis by delivering miR-216a-5p. Acta Biomater..

[34] Jiang M., He J., Sun Y., Dong X., Yao J., Gu H., Liu L. (2021). Leptin Induced TLR4 Expression via the JAK2-STAT3 Pathway in Obesity-Related Osteoarthritis. Oxid Med. Cell Longev..

[35] Zhang H.J., Liao H.Y., Bai D.Y., Wang Z.Q., Xie X.W. (2021). MAPK /ERK signaling pathway: A potential target for the treatment of intervertebral disc degeneration. Biomed. Pharmacother.

[36] Li Q., Wu M., Fang G., Li K., Cui W., Li L., Li X., Wang J., Cang Y. (2021). MicroRNA1865p downregulation inhibits osteoarthritis development by targeting MAPK1. Mol. Med. Rep..

